# Rapid and Sensitive Detection of *Yersinia pestis* Using Amplification of Plague Diagnostic Bacteriophages Monitored by Real-Time PCR

**DOI:** 10.1371/journal.pone.0011337

**Published:** 2010-06-28

**Authors:** Kirill V. Sergueev, Yunxiu He, Richard H. Borschel, Mikeljon P. Nikolich, Andrey A. Filippov

**Affiliations:** Division of Bacterial and Rickettsial Diseases, Department of Emerging Bacterial Infections, Walter Reed Army Institute of Research, Silver Spring, Maryland, United States of America; Instituto Butantan, Brazil

## Abstract

**Background:**

*Yersinia pestis*, the agent of plague, has caused many millions of human deaths and still poses a serious threat to global public health. Timely and reliable detection of such a dangerous pathogen is of critical importance. Lysis by specific bacteriophages remains an essential method of *Y. pestis* detection and plague diagnostics.

**Methodology/Principal Findings:**

The objective of this work was to develop an alternative to conventional phage lysis tests – a rapid and highly sensitive method of indirect detection of live *Y. pestis* cells based on quantitative real-time PCR (qPCR) monitoring of amplification of reporter *Y. pestis*-specific bacteriophages. Plague diagnostic phages ϕA1122 and L-413C were shown to be highly effective diagnostic tools for the detection and identification of *Y. pestis* by using qPCR with primers specific for phage DNA. The template DNA extraction step that usually precedes qPCR was omitted. ϕA1122-specific qPCR enabled the detection of an initial bacterial concentration of 10^3^ CFU/ml (equivalent to as few as one *Y. pestis* cell per 1-µl sample) in four hours. L-413C-mediated detection of *Y. pestis* was less sensitive (up to 100 bacteria per sample) but more specific, and thus we propose parallel qPCR for the two phages as a rapid and reliable method of *Y. pestis* identification. Importantly, ϕA1122 propagated in simulated clinical blood specimens containing EDTA and its titer rise was detected by both a standard plating test and qPCR.

**Conclusions/Significance:**

Thus, we developed a novel assay for detection and identification of *Y. pestis* using amplification of specific phages monitored by qPCR. The method is simple, rapid, highly sensitive, and specific and allows the detection of only live bacteria.

## Introduction

*Yersinia pestis* is a Gram-negative nonsporulating bacterium belonging to the family *Enterobacteriaceae*. *Y. pestis* is the causative agent of bubonic and pneumonic plague, a primarily zoonotic infection. The bacterium is usually transmitted from rodents and lagomorphs to humans by flea bite. Pneumonic plague is a severe infection transmissible from person to person by respiratory droplets and thought to be responsible for about 200 million human deaths during three historic pandemics. Nowadays, natural plague foci exist in Asia, Eastern Europe, Africa and both Americas, and about 2,000 cases of human plague are registered by the World Health Organization every year [1]–[3]. *Y. pestis* is classified by the CDC as a category A biothreat agent due to its easy person-to-person dissemination via aerosol, high lethality, and wide recognition as a biowarfare agent that is likely to cause mass casualties [4]. The problem is aggravated by the fact that multidrug-resistant strains of *Y. pestis* have been isolated from humans including a strain resistant to all drugs currently used for plague treatment and prophylaxis [5], [6].

In the classic clinical scenario, a confirmed diagnosis of plague includes the isolation of a pure culture of *Y. pestis* and its identification, or observing a 4-fold difference in titers of antibodies against F1 (capsule antigen) in two serum specimens from the same patient [7], [8]. This process usually takes at least 48–72 hours, which is unacceptable due to the rapid or fulminant course of plague. Therefore, numerous rapid tests for the detection of *Y. pestis* have been developed. Most of these include different variants of polymerase chain reaction, PCR [9]–[15]. Disadvantages of conventional PCR tests include the need to analyze the PCR products by gel electrophoresis and frequent contamination of the laboratory by the amplicons. Real-time PCR, allowing researchers to see an ongoing reaction by using fluorescent reporters has significantly improved the rapid detection of *Y. pestis* [16]–[26]. This approach has been successfully used for testing simulated [18], [19], [22] and actual [16] clinical specimens, environmental samples [22], food [26], and experimentally infected fleas [16], [18]. The shortest assay time described was 4 h [22], and the maximum sensitivity reached one bacterial cell per sample [18], [22], [25]. However, real-time PCR using bacterial DNA as template also has some disadvantages: it requires a preceding step of DNA extraction and cannot discriminate between live, dead and/or dormant bacteria present in diagnostic specimens.

Viruses of bacteria (bacteriophages, or phages) have been used for the diagnostics of bacterial infections since the 1930s [27]. Bacteriophage-based detection exploits fundamental properties of lytic phages: specific targeting, infection and lysis of host cells with simultaneous amplification of phage particles. Routine phage lysis assays including phage typing are still actively used for the detection and identification of various bacterial pathogens [28]–[32] but several new phage-based technologies have been developed for more rapid and efficient detection of medically important bacteria. One group of methods is based on monitoring the release of bacterial ATP [33] or such enzymes as adenylate kinase [34] or β-D-galactosidase [35] from lysed cells. Another group utilizes the indirect detection of bacteria via the propagation of specific phages engineered to express luciferase reporter genes [36]–[39], green fluorescent protein [40], or biotin ligase with subsequent biotinylation and conjugation to quantum dots [41], [42]. Finally, the technically simplest and yet efficient method of phage-based bacterial detection is the monitoring of genome amplification of a wild-type phage in the presence of a sensitive bacterium using quantitative real-time PCR (qPCR). This approach has been used first as an alternative to a standard plating test for bacteriophage λ quantitation [43] and then successfully applied for the indirect detection of *Bacillus anthracis* [44] and the plant pathogen *Ralstonia solanacearum* [45], as well as for phage-based determination of different kinds of bacterial fecal pollution of water [46].

The bacteriophage lysis test has been an integral part of *Y. pestis* detection and identification and bacteriological diagnosis of plague for about 80 years [7], [8], [47]–[50]. There are three well-studied and widely used *Y. pestis*-specific plague diagnostic phages. Two of them, ϕA1122 [7], [48], [49], [51] and the Pokrovskaya phage [47], [52]–[54], are similar to enterobacteriophage T7, display highly lytic activities and very broad lytic spectra towards *Y. pestis* strains of different origin but also lyse some strains of *Yersinia pseudotuberculosis*, the closest phylogenetic relative of *Y. pestis*. This insufficient specificity in the case of ϕA1122 can be overcome by reduction of growth temperature from 26–37°C to 20–25°C [7], [51]. ϕA1122 is recommended as an important plague diagnostic tool by the CDC [7], [49]. The third plague diagnostic phage, L-413C, is a lytic mutant of a temperate phage. L-413C is active only against *Y. pestis* (as shown on 6,000 global isolates) and rare restrictionless strains of *Escherichia coli* and does not lyse any of the 2,000 strains of *Y. pseudotuberculosis* tested [50], [52]–[54]. We have recently sequenced the genome of L-413C and shown its high homology to coliphage P2 and a mosaic structure of its tail fiber protein H, which is responsible for high specificity of L-413C [50].

The phage lysis assay usually requires the isolation of a pure *Y. pestis* culture, which takes at least 48 h. The test itself takes additional 18–24 h [7], [8]. In a recent publication, Schofield and colleagues [39] have used a fluorescently labeled ϕA1122 to monitor the early steps of its propagation and expedite the indirect detection of *Y. pestis*. A genetically modified phage was engineered to express luciferase reporter genes *luxAB* in targeted *Y. pestis* cells and allowed the detection of 820 or more bacterial cells 60 min after adding the phage. However, there was also a signal observed with 2 (of 10) *Y. pseudotuberculosis* strains and 1 (of 10) *Yersinia enterocolitica* isolates. Moreover, the prolonged incubation of the reporter phage with *Y. pestis* cells, longer than 90 min, resulted in a gradual decline in signal strength [39].

In the present study, we further explored the diagnostic capabilities of not only ϕA1122 but also L-413C and designed a simple (employing native, non-modified phages) rapid (4 h), highly sensitive (up to 10^3^ CFU/ml, equivalent to 1 cell per 1-µl sample) and specific assay for the indirect detection of live *Y. pestis* cells by qPCR monitoring of the reporter phage burst. Bacteriophage ϕA1122 provided the maximum sensitivity and displayed a significant titer rise in simulated blood specimens. This phage did not lyse any of the 17 *Y. enterocolitica* strains tested but showed some degree of amplification on 4 of the 20 *Y. pseudotuberculosis* strains tested at 28°C. The specificity of this assay was increased to practically 100% by incubating at 24°C. The L-413C-based assay was less sensitive (≥100 bacteria per sample) but more specific. We propose to use a parallel 2-phage qPCR assay for rapid and definitive identification of *Y. pestis*.

## Materials and Methods

### Ethics statement

Human blood from anonymous donors was commercially purchased from Biological Specialty Corp. (Colmar, Philadelphia). All experiments with blood artificially contaminated with *Y. pestis*, phage particles or phage DNA were performed under the human subjects use protocol approved by the Walter Reed Army Institute of Research Institutional Review Board (Protocol WRAIR #1119).

### Bacterial strains, bacteriophages and growth media

Bacterial strains used in this work are listed in Table 1. All bacteria and bacteriophage L-413C were taken from the laboratory strain collection. Phages ϕA1122, P2 *vir1*, and T7 were kindly provided by Dr. Martin E. Schriefer (Bacterial Diseases Branch, Division of Vector-Borne Infectious Disease, National Center for Zoonotic, Vector-Borne and Enteric Diseases, Centers for Disease Control and Prevention, Ft. Collins, Colorado), Dr. Richard Calendar (Department of Molecular and Cell Biology, University of California, Berkeley, California), and Dr. Ian J. Molineux (Section of Molecular Genetics and Microbiology, and Institute for Cellular and Molecular Biology, University of Texas at Austin, Austin, Texas), respectively. High-concentration stocks of ϕA1122 were prepared using attenuated *Y. pestis* strain CO92 pgm^−^ cultured at 28°C by the low multiplicity of infection method [55]. The same method was used for large-scale isolation of T7 phage but it was grown on *E. coli* C600 at 37°C. L-413C and P2 *vir1* phage stocks were prepared on *Y. pestis* CO92 pgm^−^ as described previously [50] using different temperatures of incubation, 28 or 37°C, respectively. Liquid Brain Heart Infusion (BHI) medium (Becton-Dickinson, Franklin Lakes, NJ) or BHI plates containing 1.5% Bacto Agar (Becton-Dickinson) and these with a 0.7% agar overlay were used for growing bacteria and phages. BHI for the experiments with L-413C and P2 *vir1* phages was supplemented with 1.6 mM MgCl_2_, 0.5 mM CaCl_2_, and 0.1% glucose. SM buffer [55] was used for phage storage and dilutions. All bacterial strains were grown at 28°C unless specifically indicated. Phage plaque assays were performed by the double-layer agar method as described earlier [55] with overnight incubation for L-413C and P2 *vir1* phages and 5–6 h incubation for ϕA1122. Bacterial test cultures in case of P2 *vir1* were grown at 37°C.

**Table 1:** Bacterial strains used and results of phage propagation as detected by a standard lysis procedure and by the described qPCR assay.

Species	Strain	ϕA1122 Growth/qPCR	L-413C Growth/qPCR	Species	Strain	ϕA1122 Growth/qPCR	L-413C Growth/qPCR
*Yersinia pestis*	A1122	**+**	**+**	*Y. enterocolitica*	2516-87 (O:9)	−	−
*Y. pestis*	CO92 pgm^−^	**+**	**+**	*Y. enterocolitica*	YF194 (O:18)	−	−
*Y. pestis*	KIM	**+**	**+**	*Y. enterocolitica*	YE330 (O:20)	−	−
*Yersinia pseudotuberculosis*	1 (IA)*	−	−	*Y. enterocolitica*	YF315 (O:22)	−	−
*Y. pseudotuberculosis*	IB (IB)	− (24°C)**	−	*Y. enterocolitica*	Y240 (O:25)	−	−
*Y. pseudotuberculosis*	PB1/+ (I)	− (24°C)	−	*Y. enterocolitica*	661-83 (O:27)	−	−
*Y. pseudotuberculosis*	EP2/+ (I)	−	−	*Y. enterocolitica*	YE312 (O:34)	−	−
*Y. pseudotuberculosis*	MD67 (I)	−	−	*Y. enterocolitica*	Y219 (O:44)	−	−
*Y. pseudotuberculosis*	7 (IIA)	−	−	*Y. enterocolitica*	ATCC 49397	−	−
*Y. pseudotuberculosis*	1779 (IIB)	−	−	*Yersinia aldovae*	ATCC 35237	−	−
*Y. pseudotuberculosis*	43 (III)	−	−	*Y. aldovae*	669-83	−	−
*Y. pseudotuberculosis*	III(65) (III)	−	−	*Yersinia bercovieri*	ATCC 43970	−	−
*Y. pseudotuberculosis*	MD31 (III)	−	−	*Yersinia frederiksenii*	ATCC 33644	−	−
*Y. pseudotuberculosis*	MD65 (III)	− (24°C)	−	*Y. frederiksenii*	Y225	−	−
*Y. pseudotuberculosis*	32 (IVA)	− (24°C)	−	*Yersinia intermedia*	ATCC 29909	−	−
*Y. pseudotuberculosis*	Ikegaki (IVB)	−	−	*Y. intermedia*	Y229	−	−
*Y. pseudotuberculosis*	R2 (VB)	−	−	*Yersinia kristensenii*	Y232	−	−
*Y. pseudotuberculosis*	Neilson	−	−	*Yersinia mollaretii*	ATCC 43969	−	−
*Y. pseudotuberculosis*	Hale	−	−	*Escherichia coli*	MG1655	−	−
*Y. pseudotuberculosis*	Galligue	−	−	*E. coli*	C600	−	−
*Y. pseudotuberculosis*	Parkin 413	−	−	*Shigella dysenteriae*	ATCC 13313	−	−
*Y. pseudotuberculosis*	MSU-D	−	−	*Shigella flexneri*	ATCC 9748	−	−
*Y. pseudotuberculosis*	MSU-H	−	−	*Salmonella enterica*, sv. Typhimurium	ATCC 15277	−	−
*Yersinia enterocolitica*	YE288 (O:3)	−	−	*S. enterica*, sv. Typhi	ATCC 19430	−	−
*Y. enterocolitica*	929-78 (O:6)	−	−	*Klebsiella pneumoniae*	ATCC 132	−	−
*Y. enterocolitica*	8081 (O:8)	−	−	*K. pneumoniae*	ATCC 9997	−	−
*Y. enterocolitica*	ATCC 9610 (O:8)	−	−	*Proteus vulgaris*	ATCC 6896	−	−
*Y. enterocolitica*	ATCC 23715 (O:8)	−	−	*Proteus hauseri*	ATCC 13315	−	−
*Y. enterocolitica*	ATCC 27729 (O:8)	−	−	*Citrobacter freundii*	ATCC 6879	−	−
*Y. enterocolitica*	ATCC 51871 (O:8)	−	−	*Pantoea agglomerans*	ATCC 29904	−	−
*Y. enterocolitica*	ATCC 51872 (O:8)	−	−	*Erwinia herbicola*	Lot#2 (DPG)	−	−

Notes: Phage propagation was performed at 28°C unless it is specifically indicated in the column “ϕA1122 Growth/qPCR”.

*Serovars of *Y. pseudotuberculosis* and *Y. enterocolitica* are shown in parentheses if known.

**On *Y. pseudotuberculosis* IB, a very weak propagation of ϕA1122 was observed at 24°C, six orders of magnitude lower than in *Y. pestis* CO92.

### Dynamics of *Y. pestis* lysis by bacteriophages

The phage (ϕA1122, L-413C, or P2 *vir1*) was added to an early log phase BHI broth culture of *Y. pestis* CO92 pgm^−^ (optical density at 600 nm, OD_600_ = 0.2, which corresponds to ∼1×10^8^ CFU (colony-forming units) per ml; this was repeatedly confirmed by plating) at multiplicity of infection (MOI) of 0.1 (1 bacteriophage per 10 bacterial cells). The infected culture was incubated with shaking at 200 rpm (revolutions per minute) and 28°C (or 37°C in case of P2 *vir1*), and OD_600_ was measured every 15 minutes up to 180 min. The final concentration of live bacterial cells after 3-h incubation was determined by serial 10-fold dilutions and plating on BHI agar in triplicate.

### Determination of bacteriophage burst sizes

The burst size for ϕA1122 was determined using a classic one-step growth experiment [56] modified as follows. The overnight culture of *Y. pestis* CO92 pgm^−^ was diluted 1∶50 with BHI broth and incubated at 28°C with shaking at 200 rpm to get an OD_600_ of 0.2 (∼10^8^ CFU/ml). 2.5×10^7^ PFU (plaque-forming units) of phage ϕA1122 was added in a small volume of SM buffer to 5 ml of the culture to get the MOI of about 0.05 (1∶20). At 3 min post-infection, the infected culture was diluted 500-fold (60 µl was added to 30 ml of BHI in a 500-ml flask). Immediately two zero-point samples for PFU counts (0.5 ml and 1 ml) and one sample for CFU counts (0.5 ml) were taken and the flask was placed in the incubator at 28°C with shaking at 200 rpm. The PFU determinations were done by serial 10-fold dilution of the samples without chloroform (0.5 ml) and with chloroform (1 ml plus 30 µl of chloroform) in SM buffer up to 10^−2^ and double-layer plating in triplicates. Then 1-ml aliquots were taken at 10 minute time intervals up to 120 min postinfection, treated with chloroform, serially diluted in SM buffer and plated for PFU counts. When the burst sizes of L-413C and P2 *vir1* were determined, CaCl_2_ was added to the bacterial culture up to 5 mM before adding the phage and samples for PFU counting were taken every 15 min. The experiment with P2 *vir1* was performed at 37°C.

### Phage DNA isolation

DNA of bacteriophages ϕA1122, L-413C, and P2 *vir1* for qPCR calibration purpose was extracted and purified using Lambda Midi Kit (QIAGEN Inc., Valencia, California) according to the manufacturer's protocol with the following modifications: phage particles were harvested by centrifugation in a Beckman JA-17 rotor at 13,500 rpm for 3 h and treated with proteinase K at 55°C for 1 h for better disruption of phage capsids. Purity of DNA preparations was confirmed by agarose gel electrophoresis. DNA concentrations were measured by using NanoDrop ND-1000 spectrophotometer (Thermo Fisher Scientific Inc., Waltham, Massachusetts).

### Phage propagation for qPCR assays

Overnight broth bacterial cultures were diluted 1∶100 in BHI and grown with shaking until their concentration reached approximately 10^8^ CFU/ml. Phage was added to different 10-fold dilutions of bacteria at final concentration of 10^5^ PFU/ml to minimize the impact of the initial amount of phage on qPCR results. The infected bacterial culture was incubated with shaking for 3 h at 28°C. To prevent a non-specific low-level propagation of ϕA1122 on some strains of *Y. pseudotuberculosis* (see Table 1), they were incubated at 24°C. One milliliter aliquots were taken at certain time points and 30 µl of chloroform was immediately added to each sample to kill bacterial cells and release phage particles. One microliter of such a mixture (lysate) was directly used as target bacteriophage DNA for qPCR analysis without any DNA isolation step.

### Phage amplification in simulated clinical samples containing *Y. pestis*

Simulated clinical tests were done with EDTA-treated whole human blood (Biological Specialty Corp.). The blood was diluted 10-fold with BHI broth (such dilutions are routinely used in bacteriological analysis of blood for plague [8]) containing log-phase culture of *Y. pestis* CO92 pgm^−^ at concentrations ranging from 10^8^ to 10^3^ CFU/ml. Phage ϕA1122 was added to the final concentration of 10^5^ PFU/ml. One milliliter aliquots were taken every 60 min during 5 h, and 30 µl of chloroform was added. Each sample was diluted 20-fold with SM buffer to minimize the inhibitory effect of blood on qPCR, phage plating was performed for PFU counts and 1 µl of each dilution was used for qPCR reaction. The phage DNA extraction step was omitted.

### Primer design

Primers for qPCR monitoring of phage amplification were designed by using Beacon Designer™ program (Premier Biosoft Int., http://www.premierbiosoft.com). The target for the ϕA1122-based assay was the RNA polymerase gene, and primers were selected from its variable sequences to minimize potential cross-reactions with relative T7 and other T7-like phages. The primers for L-413C were selected from the unique tail fiber gene H having a mosaic structure [50]. Several pairs of primers with lowest self- and cross-complementarity were checked in qPCR with 100 PFU of ϕA1122 or L-413C (without a DNA extraction step), and two pairs providing the highest amplification signals were selected for further work (Table 2). The primers were analyzed using BLAST (Basic Local Alignment Search Tool) engine at the NCBI web site (http://blast.ncbi.nlm.nih.gov) against the nonredundant (nr) database to make sure that at least one primer from the pair represented a unique nucleotide sequence. The primers were also tested for specificity against *Y. pestis* genomic DNA (suspension of strain CO92 pgm^−^ in distilled water boiled for 5 min; about 5×10^8^ CFU/ml) and against the closest relatives of phages ϕA1122 (T7) and L-413C (P2). Primers for L-413C DNA amplification were specific for this phage and did not amplify DNA of P2 *vir1*. Despite the fact that ϕA1122 and T7 RNA polymerase genes share 93% nucleotide sequence identity, no cross reaction of ϕA1122 primers was observed after 40 cycles of qPCR with 10^3^ PFU of T7. However, a weak cross reaction (Ct = 28.37) was observed with a high dose of T7, 10^6^ PFU, although the reaction with 10^6^ PFU of ϕA1122 was much more robust (Ct = 7.50).

**Table 2:** Primers used for qPCR in this study.

Designation	DNA Sequence	Length (bp)	Tm (°C)	GC%	Product (bp)
ϕA1122-F	5′-CCAAATGGAAGCACTGCCCTGTAG-3′	24	61.8	54.2	105
ϕA1122-R	5′-ATGCGGTGAGAGCCTCAGGATTC-3′	23	62.1	56.5	
L-413C-F	5′-ACGTGGTCATGTCCGTCACAATC-3′	23	60.9	52.2	75
L-413C-R	5′-CAGAACCCCATTGCCTTTATCTTCAG-3′	26	60.3	46.2	

### Quantitative real-time PCR assay

Maxima™ SYBR Green/ROX qPCR Master Mix 2× (Fermentas Inc., Glen Burnie, Maryland) was used in all qPCR experiments according to the vendor's recommendations. Reactions were performed in a total volume of 20 µl containing 1 µl of DNA template (pure phage DNA or lysates containing live phage particles), 10 µl of the master mix, and 0.9 µM of each primer and were run on a LightCycler 2.0 (Roche Applied Science, Indianapolis, Indiana). The cycling parameters were: 95°C, 10 min; 40× (95°C, 20 s; 60°C, 60 s) with fluorescence measurement at the end of each cycle.

### qPCR sensitivity test

*Y. pestis* CO92 pgm^−^ was grown in BHI broth at 28°C until OD_600_ reached 0.2. The culture was serially diluted from 10^8^ CFU/ml to 10^3^ CFU/ml. Each bacterial suspension was infected with ϕA1122 or L-413C at final concentration of 10^5^ PFU/ml (100 PFU/µl). 1-ml aliquots from each culture were collected every 30 minutes for ϕA1122 or every 60 minutes for L-413C within 3 h post-infection, treated with chloroform (30 µl), and 1µl was used for qPCR.

### Evaluation of inhibitory effect of blood on qPCR

Whole human blood (Biological Specialty Corp.) and dilutions of 1∶10 and 1∶20 with SM buffer were tested. Purified DNA of ϕA1222 was added at concentrations ranging from 0.5 ng/µl to 0.5 fg/µl, and the qPCR reactions were run in duplicate. Phage DNA solutions in SM buffer at the same concentrations were used as a negative control.

### Statistical analysis

Results of all qPCR tests of phage amplification are presented as the mean values of three independent experiments. Statistical significance was determined by One Way ANOVA (analysis of variance) analysis using a free online program at the web site of Vassar College (http://faculty.vassar.edu/lowry/anova1u.html). *P* values <0.05 were considered significant.

## Results

### Lytic activities against *Y. pestis* and propagation rates of three bacteriophages

**Figure 1:**
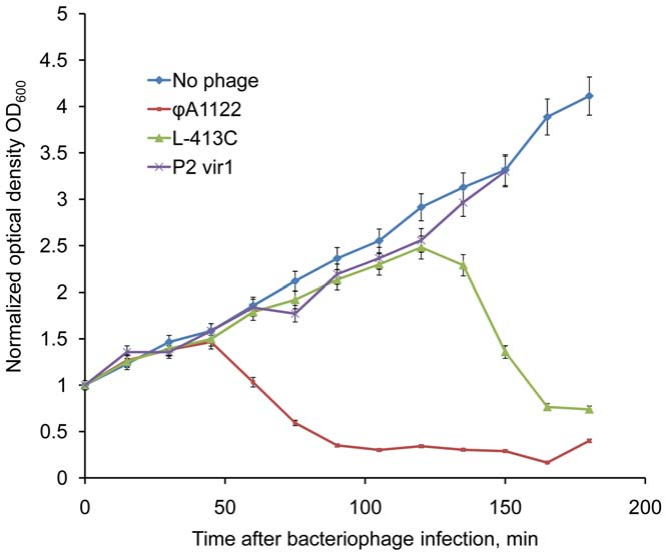
Lytic properties of bacteriophages ϕA1122, L-413C, and P2 *vir1* towards *Y. pestis* CO92 pgm^−^. The dynamics of lysis was determined in BHI broth at multiplicity of infection of 0.1. Optical density was normalized to the start of infection (1 on the Y axis corresponds to the initial OD_600_ = 0.2).

To develop a phage-based qPCR system for *Y. pestis* detection, we tested plague diagnostic phages ϕA1122 [7], [48], [49], [51] and L-413C [50], [52]–[54], as well as a clear plaque mutant of coliphage P2, P2 *vir1* [57]. We have previously shown that P2 *vir1* lyses *Y. pestis* at 37°C, has relatively low plaquing efficiency at 28°C, but is not active against a wild-type strain of *Y. pseudotuberculosis* at either temperature [50]. Since the speed of phage propagation is critical for qPCR efficiency, we first checked some parameters of amplification of ϕA1122, L-413C, and P2 *vir1* on *Y. pestis* CO92 pgm^−^. Phage propagation rates can be indirectly measured by their lytic activity. The plaque sizes of the three phages after 24-h incubation at 37°C were measured. This temperature of growth was chosen to enhance lytic properties of P2 *vir1* and standardize the conditions (ϕA1122 and L-413C at 28°C appeared identically). The mean diameters of plaques (measured for 20 plaques) for ϕA1122, L-413C, and P2 *vir1* were 10, 3, and 1 mm, respectively. Dynamics of bacterial lysis by each of the three phages in BHI broth was then measured at 28°C, the optimal temperature for *Y. pestis* growth (Fig. 1). In case of ϕA1122, markedly detectable lysis occurred 45 minutes after infection. In contrast with this, clarification of culture suspension infected with L-413C occurred only in 2 h but was vigorous. No lysis of *Y. pestis* culture infected with P2 *vir1* was observed at 28°C. It is important that ϕA1122 efficiently killed *Y. pestis* cells during its propagation; we were unable to detect any viable bacterial cells after 1-h incubation with ϕA1122.

**Figure 2:**
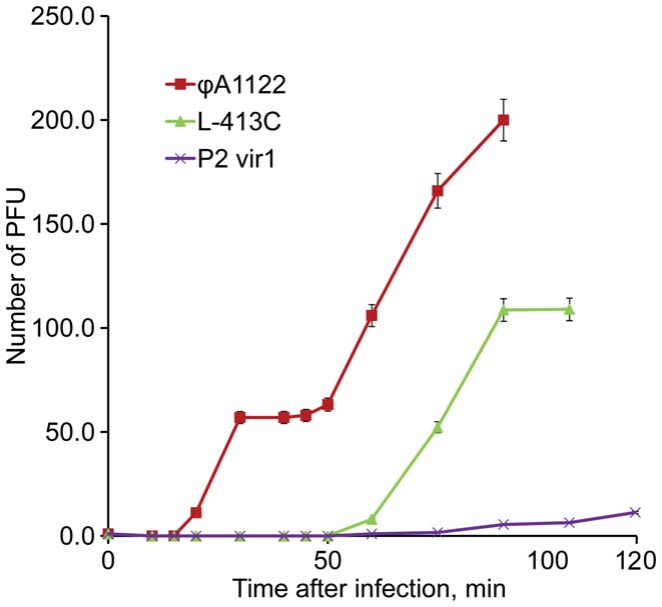
Determination of lysis speed and burst sizes for bacteriophages ϕA1122, L-413C, and P2 *vir1* on *Y. pestis* CO92 pgm^−^. Phage burst sizes (an average phage progeny produced by one bacterial cell) correspond to plateaus on the curves.

Phage propagation rates were also estimated more directly by determination of burst size (Fig. 2), which is the number of phage particles released from a single host cell. The burst sizes of ϕA1122, L-413C, and P2 *vir1* for *Y. pestis* CO92 pgm^−^ were about 57, 115, and 9 PFU, respectively (see the plateaus on the phage growth curves in Fig. 2). The lengths of lytic cycles of these three phages were approximately 30, 90, and 90 min. Thus, L-413C has the highest burst size, about 115 particles from one *Y. pestis* cell, but its lytic cycle is relatively long, approximately 90 min. Phage ϕA1122 has a lower burst size, 57 PFU, but in 90 min a single phage is supposed to make three lytic cycles and produce, under ideal conditions, 57^3^≈1.9×10^5^ phages. Thus, ϕA1122 showed the maximum lytic activity and the highest propagation rate; P2 *vir1* had low lytic activity and was slow growing; and L-413C had intermediate growth characteristics. Due to the comparatively slow and weak amplification of P2 *vir1* on *Y. pestis*, this phage was excluded from further experiments.

### Performance testing of qPCR with purified DNA from *Y. pestis*-specific phages and with intact phage particles

**Figure 3:**
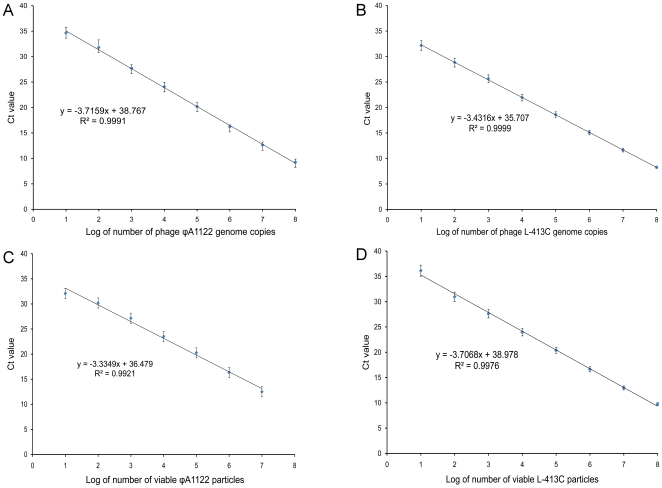
Parameters of ϕA1122- and L-413C-based qPCR tests for phage DNA and live phage particles determined by linear regression method. A and B, standard curves plotted for DNA concentrations of ϕA1122 and L-413C, respectively. C and D, standard curves plotted for live phage particles of ϕA1122 and L-413C, respectively.

The quantitative parameters of phage-based qPCR were first tested by using serial ten-fold dilutions of DNA purified from ϕA1122 and L-413C. These tests were performed in triplicate and yielded a log linear relationship between DNA concentrations and threshold cycle number (Ct), spanning an 8-log dilution series, from 5 ng down to 0.5 fg of DNA (Fig. 3, A and B). We calculated that this corresponds to 12 to 1.2×10^8^ genome equivalents of ϕA1122 and 15 to 1.5×10^8^ genome equivalents of L-413C based on the facts that their genome sizes are 37,555 [49] and 30,728 bp [50], respectively. To compare qPCR results obtained using purified DNA and intact phage particles, a series of 10-fold phage lysate dilutions was prepared in SM buffer, and qPCR reactions were run on different concentrations of phage particles ranging from 10^1^ to 10^7^ PFU per 1 µl of phage lysate (per 20 µl of qPCR sample) for ϕA1122 and from 10^1^ to 10^8^ PFU for L-413C. The number of viable particles per sample was confirmed by plaque assays. The results of qPCR with the particles presented in Fig. 3 (C and D) are in accordance with the data obtained with phage DNA (Fig. 3, A and B). Phage lysates treated with DNase I showed the same results as untreated phage particles (data not shown).

### Sensitivity of phage-based qPCR detection of *Y. pestis*

**Figure 4:**
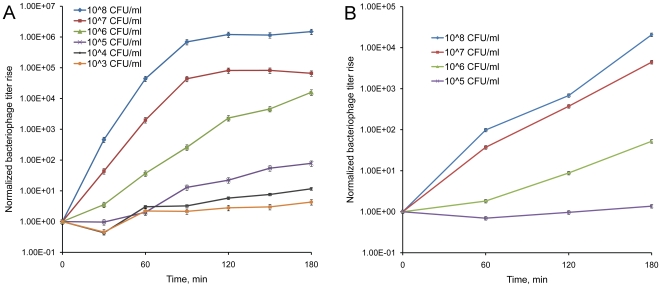
Dynamics of growth of phages ϕA1122 and L-413C on different concentrations of *Y. pestis* cells detected by qPCR. The starting points of phage infection correspond to 100 PFU per 1 µl sample and are normalized to 1. A. The titer rise of ϕA1122. B. L-413C amplification.

To determine the sensitivity of phage-mediated detection of *Y. pestis*, we conducted a series of experiments where reporter phages ϕA1122 and L-413C were propagated on various concentrations of *Y. pestis* CO92 pgm^−^ cells with subsequent PFU counts and qPCR tests. The phage titer rises determined by qPCR (Fig. 4) were calculated based on Ct values and our calibration data (Fig. 3). The starting points of infection corresponding to 100 PFU per 20-µl sample (containing 1 µl of phage lysate) were normalized to 1 (Fig. 4). Our data showed that the detection of *Y. pestis* using ϕA1122 is robust and there are marked differences in phage yields at different concentrations of *Y. pestis*. A significant rise in phage concentration and qPCR signal was observed even at the lowest possible starting concentration of *Y. pestis*, 10^3^ CFU/ml, or one bacterium per 20 µl of qPCR sample (about 50-fold, see Fig. 4, A). This indicates that the limit of ϕA1122-based qPCR detection of *Y. pestis* was 10^3^ CFU/ml, equivalent to one host bacterium per 20 µl of qPCR sample (or per 1 µl of phage lysate). At the same time, a rise in L-413C titer and the qPCR signal was statistically significant only at higher concentrations of host cells, 10^8^–10^5^ CFU/ml that corresponds to 10^5^–10^2^ CFU in 20 µl of qPCR sample (Fig. 4, B). Thus, the sensitivity of L-413C-mediated qPCR assay was 10^5^ CFU/ml, or 100 CFU per sample. The entire detection test included 3-h phage propagation and 1-h qPCR reaction and took 4 h.

### Specificity of qPCR assays

The specificity of phage-based detection was tested on 62 strains belonging to 19 bacterial species of *Enterobacteriaceae* including nine *Yersinia* species (Table 1). Both the ability of ϕA1122 and L-413C to propagate on various bacteria at 28°C and the potential rise in qPCR signal intensity were studied. L-413C did not form any plaque and did not cause any decrease in Ct value on bacterial cultures other than *Y. pestis*, confirming the high specificity of this phage towards *Y. pestis* [50], [52], [53]. Both propagation and qPCR tests of ϕA1122 incubated at 28°C were negative with nonpathogenic *Yersinia*, as well as with each of 17 *Y. enterocolitica* strains. However, bacteriophage ϕA1122 grew on 4 out of 20 different strains of *Y. pseudotuberculosis* (the closest phylogenetic relative of *Y. pestis*), and the phage propagation was easily detected by qPCR analysis. To enhance the specificity, we reduced the incubation temperature during ϕA1122 infection from 28°C to 24°C [7], [51]. This allowed the assay to get practically 100% specificity: only a low-level amplification of ϕA1122 was observed on one strain of *Y. pseudotuberculosis*, IB (the phage yield was 10^6^ lower than that determined for *Y. pestis* CO92). The same result was obtained after propagation of ϕA1122 at 20°C.

### Simulated clinical tests

**Figure 5:**
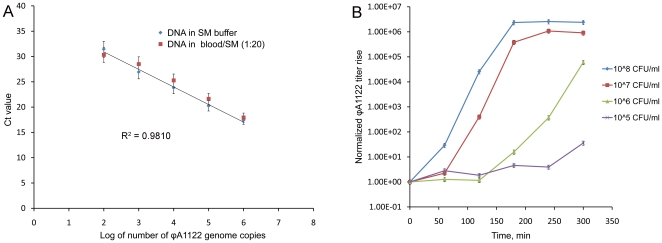
qPCR tests on simulated clinical human blood samples. A. Linear regression of ϕA1122 DNA concentration in blood diluted 1∶20 in comparison with SM buffer data. B. ϕA1122-based detection of *Y. pestis* in artificially contaminated blood diluted 10-fold with BHI broth. To calculate the actual bacterial loads in the undiluted blood samples, the CFU numbers shown should be multiplied by 10. The starting points of phage infection correspond to 100 PFU per 1 µl sample and are normalized to 10^0^ = 1.

Comparison of qPCR efficiencies for detection of ϕA1122 in EDTA-treated human blood diluted 1∶20 and in SM buffer showed virtually no inhibition of the reaction by blood components (Fig. 5, A). To evaluate the possibility of using phage-based qPCR for diagnostics of human plague, we performed ϕA1122 propagation and qPCR analysis in human blood experimentally contaminated with *Y. pestis* (Fig. 5, B). Phage ϕA1122 propagated in blood diluted with BHI broth 1∶10, although slower than on the control culture grown in BHI, and this propagation was detected by qPCR. A statistically significant rise in phage titer was observed after 5 h at the lowest concentration of *Y. pestis* of 10^5^ CFU/ml in diluted blood, which corresponded to 100 CFU in a qPCR sample and 10^6^ CFU/ml in the undiluted whole blood sample.

## Discussion

We report the development of a novel quantitative real-time PCR assay for indirect detection of *Y. pestis* based on amplification of non-modified reporter *Y. pestis*-specific bacteriophages in the presence of the host bacteria. This qPCR method utilizing phages ϕA1122 [7], [48], [49], [51] and L-413C [50], [52]–[54] is a reasonable alternative to a standard phage lysis test. It was shown to be easy, reliable, rapid, highly sensitive and specific. Since bacteriophages propagate only on viable and culturable cells [40], [58], our approach allows detection of live and metabolically active cells of *Y. pestis*, which may be specifically important in forensic studies and for characterization of activity of natural plague foci. qPCR with primers specific for phage DNA has been previously used for quantitation of bacteriophage λ instead of routine plating assay [43] and then employed for phage-based detection of several bacterial species [44]–[46]. In case of the use of gamma phage for indirect detection of *B. anthracis*, the assay took only about 5 h, its sensitivity reached one bacterial cell per sample, and amplification was not observed with four other species of Gram-positive and Gram-negative bacteria [44].

We analyzed three bacteriophages capable of lysing *Y. pestis*, ϕA1122, L-413C, and P2 *vir1* [57], as potential reporters for qPCR phage-based detection of the bacterium. Since the developed assay is based on the detection and quantitation of rapidly replicating phage DNA, we first studied some parameters of lysis and propagation rates of the three phages including mean diameters of plaques, dynamics of bacterial lysis, burst size and duration of lytic cycle. Plague diagnostic bacteriophages ϕA1122 and L-413C displayed high lytic activities and propagation rates on *Y. pestis*, particularly ϕA1122 (Fig. 1 and 2). Phage ϕA1122 formed the largest plaques, had the shortest time of the lytic cycle and caused the fastest and most robust lysis of the bacterial culture. Importantly, ϕA1122 efficiently inactivated *Y. pestis* in all diagnostic samples, which is advantageous when working with this infectious agent. Of the three phages studied, L-413C showed the biggest burst size, a longer lytic cycle and delayed but vigorous lysis of bacterial cells. P2 *vir1* displayed low lytic activity, slow and weak growth on *Y. pestis* and thus was excluded from further tests. These data were essential for designing our diagnostic assay, but also can be used for future bacteriophage plague therapy applications [59] and add some knowledge to the biology of phages ϕA1122, L-413C, and P2 *vir1*.

Testing of the quantitative parameters of phage-based qPCR with purified DNA of phages ϕA1122 and L-413C showed reliable standard curves down to 0.5 fg (12–15 genome equivalents) that, based on burst sizes, corresponds to 0.1–0.2 cells of *Y. pestis*. The calculated numbers of phage genome equivalents correlated well with the standard curves based on qPCR with intact phage particles (Fig. 3). Multiple experiments on the propagation of a standard number of ϕA1122 and L-413C particles on various concentrations of *Y. pestis* cells with subsequent plaque counts and qPCR tests showed that the limit of ϕA1122-mediated detection of *Y. pestis* was as low as 10^3^ CFU/ml, or one bacterium per qPCR sample. The sensitivity of L-413C-based test was 10^5^ CFU/ml, or 100 CFU per sample (Fig. 4), and the duration of the whole procedure in both cases was 4 h. These results were obtained without the concentration of samples or DNA purification, which is a standard step of conventional real-time PCR assays targeting bacterial genes [60]. Several groups have reported on the use of different variants of real-time PCR targeting plasmid and chromosomal genes of *Y. pestis* [16]–[26]. The best detection limit observed in these studies also reached one bacterial cell per sample [18], [22], [25] and the shortest assay time described was the same as in our work, 4 h [22].

The use of simplex real-time PCR targeting a single plasmid [16], [17], [19] or chromosomal [21] gene can result in missed detection of mutant strains and the entire biovars of *Y. pestis*. For example, enzootic strains of *Y. pestis* isolated from voles in Transcaucasian Highland and Mountain Dagestan are missing the pPst plasmid [61], the most prevalent target for diagnostic PCR. The loss from laboratory strains of *Y. pestis* of one, two, or three plasmids [61], or the extensive chromosomal pigmentation region [62] can occur, and some strains cured of the pPst [63]–[65] or pFra [63], [66], [67] plasmid maintain full virulence. Real-time PCR in multiplex format [18], [22]–[26] broadens the spectra of detected strains but this makes the reaction more technically complicated. Based on the facts that ϕA1122 [7], [49], [51] and L-413C [50], [52], [53] can lyse almost all (99.8–99.9%) *Y. pestis* strains of several thousand tested, we propose a broad-range qPCR method in a simplex format.

The specificity of this phage-based detection procedure was tested on 62 strains of 19 bacterial species including a variety of isolates of pathogenic and non-pathogenic *Yersinia*. Both the ability of ϕA1122 and L-413C to propagate on various bacterial cultures and phage qPCR signals in the presence of the bacteria were determined (Table 1). Bacteriophage ϕA1122 has been shown to lyse practically all strains of *Y. pestis* and some isolates of *Y. pseudotuberculosis* at 26–28°C and higher temperatures [7], [49], [51]. This issue can be bypassed by using lower temperatures for growth, 20–25°C [7], [51]. In our experiments, ϕA1122 grew at 28°C on 20% (4 of 20) strains of *Y. pseudotuberculosis*, and the phage amplification was registered by qPCR. The reduction of growth temperature to 24°C allowed us to reach virtually 100% specificity: only one *Y. pseudotuberculosis* strain (IB) displayed a low degree of ϕA1122 propagation and a weak qPCR signal (the propagation rate was six orders of magnitude lower than that obtained on *Y. pestis* CO92). The same weak growth of ϕA1122 was observed at 20°C. All ϕA1122 propagation and qPCR tests were negative with 17 *Y. enterocolitica* strains and 9 isolates of nonpathogenic *Yersinia*. The L-413C assay did prove to be 100% specific when using the regular temperature of incubation, 28°C: the amplification of this phage was detected by using plaque counts and qPCR only on *Y. pestis* (Table 1). Our data confirmed the high specificity of L-413C to *Y. pestis* [50], [52], [53]. Inter-laboratory trials of L-413C on 6,000 global isolates of *Y. pestis* and 2,000 strains of *Y. pseudotuberculosis* have shown an absolute specificity of this phage for the plague agent [53]. Based on this extraordinary specificity of L-413C confirmed in our tests, we propose to use parallel qPCR assays with both phages for rapid and reliable detection and identification of *Y. pestis*.

The clinical performance of this phage-based qPCR assay was evaluated with EDTA-treated human blood artificially contaminated with *Y. pestis*. The appropriate dilution of blood with SM buffer (1∶20) allowed us to avoid inhibition of ϕA1122-specific qPCR by blood components (Fig. 5, A) but this decreased the assay sensitivity twenty-fold. Phage ϕA1122 was shown to propagate in the spiked blood diluted 1∶10 with BHI by the methods of plating and qPCR. Such dilutions with a nutrient broth are typically done in bacteriological tests of blood for plague [8]. Statistically significant phage amplification was detected in 5 h, and the detection limit was 100 CFU of *Y. pestis* in a qPCR sample, which corresponded to 10^5^ CFU/ml in diluted blood (Fig. 5, B) and to 10^6^ CFU/ml in undiluted blood. It has been previously shown that the efficiency of *Y. pestis* detection by real-time PCR from blood of infected nonhuman primates is lower compared with sera and especially with swabs from the oropharyngeal cavity [16]. The detection threshold observed by those authors was 2.1×10^5^ copies of a gene located in the pPst plasmid [16] known to have 186 copies per genome [68]; this makes the detection limit 1,129 CFU, which is higher than in our tests. Other investigators achieved lower detection limits for real-time PCR assays with simulated human respiratory specimens, but only after multi-step sample treatment [19], [22]. For example, a detection limit of 85 CFU was observed after the pretreatment of sputum with a mucolytic agent, centrifugation, resuspending in TE buffer, artificial contamination with *Y. pestis*, and DNA extraction [22]. This sensitivity level of our test with spiked blood was satisfactory without pretreatment because colony counts in blood cultures of plague patients can reach 4×10^7^ CFU/ml [69]. The sensitivity level could be enhanced using a concentration step.

Bacteriophages have been used for *Y. pestis* detection and plague diagnosis since the early 1930s [7], [8], [47]–[50]. ϕA1122 is recommended as an essential plague diagnostic tool by the CDC [7], [49], and L-413C is routinely used for the same purpose in many countries of Eastern Europe and Central Asia [50], [52]–[54]. Most phage plating assays require the isolation of a pure culture of *Y. pestis*. The culture isolation together with the lysis test usually takes three days [7], [8]. A genetically engineered phage ϕA1122 expressing luciferase reporter genes has been recently used for indirect detection of *Y. pestis* [39]. The method was shown to be very rapid (1 h) and allowed detection of ≥820 *Y. pestis* cells but fluorescent signals higher than the background were observed with two *Y. pseudotuberculosis* strains and even with a *Y. enterocolitica* isolate. An additional concern about this method is the short time frame for effective application: a gradual decline in signal strength has been found when using an incubation time of the phage with *Y. pestis* longer than 90 min [39].

We propose qPCR with the use of both ϕA1122 and L-413C as a reasonable alternative to routine phage lysis tests for detection and identification of *Y. pestis*. Our assay is simple (because it utilizes native, non-modified phages; this is also important for possible expanding the panel of phages used), rapid (4 h), highly sensitive (up to 1 cell per sample) and specific for *Y. pestis*. This method can be used for plague diagnostics, forensic purposes and the monitoring of plague foci. Another potential application is for pharmacokinetics studies and the evaluation of phage propagation *in vivo* during bacteriophage therapy. Such applications are important due to the emergence of multidrug-resistant strains of *Y. pestis* [5], [6].
